# Two Cysts of Unusual Origin

**Published:** 1894-09

**Authors:** Charles A. Morton

**Affiliations:** Surgeon to the Bristol General Hospital; Demonstrator of Anatomy, University College, Bristol


### TWO CYSTS OF UNUSUAL ORIGIN.

BY

CHARLES A. MORTON, F.R.C.S. Eng.,

*Surgeon to the Bristol General Hospital; Demonstrator of Anatomy, University College, Bristol.*

### I.

### IMPLANTATION CYST.

IMPLANTATION cysts (as Bland Sutton calls them) are tumours caused by the implantation of a portion of epidermis into the subcutaneous tissue, where it grows into a cyst, resembling a dermoid. They always result from injury. Similar cysts are sometimes formed in the eye from implantation of an eyelash or portion of corneal epithelium. The nature of these cysts is fully described by Bland Sutton in his recent work on Tumours.

A woman, 34 years of age, came to see me as an out-patient at the General Hospital, with the following history. When a little girl she ran a thorn into her thigh, and ever since there had been a minute opening where the thorn perforated. During the last year a hard swelling had formed around this, which had given her a good deal of pain. There was a perfectly circular opening in the skin the size of a sixpenny piece, with smooth thin edges, through which could be felt a black hard mass, with a minute central depression in it, which extended some distance under the surrounding skin. This condition had only been present for a year. On slitting up the opening the black mass easily shelled out. It was then seen to have been contained in a thin-walled cyst, which was without much difficulty dissected out. Its inner surface was white and shining. On making a section of the black mass, the centre was seen to be amber coloured with a still more central depression in it, about the size and shape of a small needle's point, evidently where the thorn had been. The mass was so hard it could only be cut with a knife by using great force. After being in spirit a few days a very remarkable change took place in it. It greatly increased in size, softened, and split up into innumerable laminæ, like the layers of an onion.[1] Microscopically the structure was that of corneous epithelium.

There are a few of these cysts described in the *Transactions of the Pathological Society*. They are called sebaceous or dermal cysts. One of the first recorded is by Mr. Poland.[2] It followed a puncture with a piece of steel. Its wall was made up of densely-packed laminæ of squamous epithelial scales, resembling very much the structure of the mass in the interior of my cyst, but there was no cyst-wall. Bowlby records one due to pricking the finger with a needle,[3] and Barker[4] one after amputation of the finger. The structure of such cysts is

[1] The specimen was shown at the Bristol Medico-Chirurgical Society.

[2] *Tr. Path. Soc.*, vol. xxxv., 1884, p. 399. [3] *Ibid.* [4] *Ibid.*, vol. xxxvii., 1886, p. 478.

beautifully shown in the plate which accompanies Barker's paper. Similar cases are also recorded by Stabb[1] and Raymond Johnson.[2]

In my case, the length of time which elapsed before signs of the cyst were apparent is very remarkable. The thorn doubtless carried some surface epithelium into the subcutaneous tissue, and this developed into the cyst. An analogous condition is seen in the cases recorded by Poland and Bowlby, and in the instances, referred to by Bland Sutton, in which similar cysts have developed in cattle, presumably from prodding with metal-pointed sticks.

In contrast with this form of tumour, the following case, also one of a cyst following injury, will be of interest.

### II.

### CYST FORMED AROUND A FOREIGN BODY.

A boy, aged 13, came to my out-patient room on April 10th, 1893, with a swelling in the hand, and the following history. During the previous June he had fallen off a wall and cut his hand against a stone. He went to a doctor, who examined it and told his mother to poultice it in case it contained any foreign body. The wound healed in a week, but a little swelling remained, which gave him slight pain when he played cricket, but only at such times. During the last three weeks, however, it had steadily increased in size and caused him considerable pain on moving his hand in writing. Over the centre of the annular ligament was a round soft swelling with a very hard irregular centre, lying under a scar in the skin. When pressure was made on the hard body it pricked him. The soft swelling was a fibrous cyst with a smooth inner surface containing a little serous fluid, in which was lying a piece of glass half an inch in diameter. After the removal of the glass the thickening caused by the cyst-wall almost completely disappeared, and he could use his hand quite well. This was a cyst formed from condensation of the surrounding tissues from irritation of the foreign body. It is remarkable how long the glass lay embedded without causing discomfort, and the complete ignorance on the

[1] *Tr. Path. Soc.*, vol. xlii., 1891, p. 314. [2] *Lancet*, 1892, vol. ii., p. 1048.

part of the boy that there had been any glass about when he cut his hand is very surprising. Perhaps so long as the glass was firmly embedded no pain was caused; but as soon as a space containing fluid was formed around it movement became possible, and hence the pain. The pain and rapid increase in size certainly went together, the latter indicating probably the cyst formation.

